# Racial and Ethnic Disparities in Mother’s Milk Provision Among Mothers of Preterm Infants

**DOI:** 10.1001/jamanetworkopen.2025.10781

**Published:** 2025-05-16

**Authors:** Nikita S. Kalluri, Erika G. Cordova-Ramos, Sunah S. Hwang, Katherine R. Standish, Margaret G. Parker

**Affiliations:** 1 Division of Neonatology, Department of Pediatrics, UMass Chan Medical School, Worcester, Massachusetts; 2 Division of Newborn Medicine, Boston Children’s Hospital, Boston, Massachusetts; 3 Department of Pediatrics, Boston University Chobanian & Avedisian School of Medicine, Boston, Massachusetts; 4 Department of Neonatology, University of Colorado School of Medicine, Aurora; 5 Department of Family Medicine, Boston University Chobanian & Avedisian School of Medicine, Boston, Massachusetts

## Abstract

**Importance:**

The extent of mother’s milk provision by race and ethnicity and its change over time is poorly understood in the US preterm population.

**Objective:**

To examine US prevalence and trends over time of mother’s milk initiation and continuation at 12 weeks after birth (2009-2019) by maternal race and ethnicity and to examine associations of maternal race and ethnicity and mother’s milk initiation and continuation among mothers of preterm infants.

**Design, Setting, and Participants:**

This cross-sectional study used data from the nationally representative Pregnancy Risk Assessment Monitoring System database. Participants included mothers of preterm infants identifying as non-Hispanic Asian, non-Hispanic Black, Hispanic any race, or non-Hispanic White, with survey completion 12 or more weeks post partum from 2009 to 2019. Data were analyzed from February 2022 to June 2024.

**Exposure:**

Maternal race and ethnicity obtained from birth certificate data.

**Main Outcomes and Measures:**

Mother’s milk initiation and continuation at 12 weeks were assessed using survey questions. The overall prevalence and trends over time by maternal race and ethnicity were determined. Multivariable regression was used to analyze independent associations between maternal race and ethnicity and mother’s milk initiation and continuation.

**Results:**

Among 1 523 131 (weighted) mother-preterm infant dyads, mother’s milk initiation increased significantly from 2009 to 2019 for White and Black mothers but not for Asian or Hispanic mothers. Mother’s milk provision at 12 weeks increased significantly among all groups. There were significant differences in mother’s milk outcomes between groups. Initiation was highest for Asian mothers (92.8%; 95% CI, 91.1%-94.4%), followed by Hispanic (88.1%; 95% CI, 86.5%-89.8%), White (84.1%; 95% CI, 83.3%-84.9%), and Black (75.3%; 95% CI, 73.4%-76.6%) mothers. After adjusting for covariates of interest, compared with White mothers, initiation remained higher for Asian (adjusted relative risk [aRR], 1.09; 95% CI, 1.06-1.12) and Hispanic (aRR, 1.10; 95% CI, 1.08-1.12) mothers. Continuation at 12 weeks was highest for Asian mothers (65.4%; 95% CI, 62.7%-68.0%), followed by Hispanic (48.2%; 95% CI, 46.1%-50.3%), White (47.7%; 95% CI, 46.7%-48.7%), and Black (34.3%; 95% CI, 32.9%-35.7%) mothers. After adjustment, continuation was higher for Asian (aRR, 1.37; 95% CI, 1.24-1.47) and Hispanic (aRR, 1.33; 95% CI, 1.27-1.41) mothers compared with White mothers.

**Conclusions and Relevance:**

In this cross-sectional study, mother’s milk provision among preterm infants increased from 2009 to 2019, but notable racial and ethnic disparities persist. Future work should address barriers to continued provision of mother’s milk for preterm infants.

## Key Points

**Question:**

How many mothers of preterm infants initiated and continued providing mother’s milk for 12 weeks after birth, and were there differences by race and ethnicity?

**Findings:**

In this cross-sectional survey representing 1 523 131 mothers of preterm infants from 2009 to 2019, mother’s milk initiation and continuation differed by race and ethnicity, with initiation increasing significantly for White and Black mothers but not for Asian or Hispanic mothers and continuation increasing in all groups. After adjustment, compared with White mothers, Black mothers had similar rates, whereas Asian and Hispanic mothers had higher initiation and continuation rates.

**Meaning:**

These finding suggest that mother’s milk provision among preterm infants increased from 2009 to 2019 but racial and ethnic disparities persist; barriers to continued provision of mother’s milk for preterm infants should be identified and addressed.

## Teaser

This cross-sectional study examines the prevalence and trends over time of mother’s milk initiation and continuation at 12 weeks after birth from 2009 to 2019 by maternal race and ethnicity and the associations of maternal race and ethnicity and mother’s milk initiation and continuation.

## Introduction

Infants born preterm (<37 weeks’ gestational age) comprise approximately 10% of births in the US, with recently increasing rates.^1^ Mother’s own milk is the optimal source of nutrition for preterm infants and has been associated in a dose-dependent manner with protection against morbidities of prematurity, including necrotizing enterocolitis, late-onset sepsis, and adverse neurodevelopment, as well as fewer rehospitalizations.^2,3,4,5,6^ It also provides targeted protective proteins and antibodies throughout the first months of life.^5^ Therefore, professional organizations including the Centers for Disease Control and Prevention (CDC) and American Academy of Pediatrics recommend mother’s milk for preterm infants and promote evidence-based lactation support practices unique to mother–preterm infant dyads.^7^ In the past 15 years, enormous local and state efforts have aimed to improve the provision of mother’s milk, particularly during the first 3 months, when many preterm infants experience prolonged hospitalization and are at greatest risk for medical complications of prematurity.^8,9,10,11,12^

National surveillance of full-term infants has highlighted stark racial and ethnic breastfeeding disparities, where non-Hispanic Black mothers have lower rates than non-Hispanic White and Hispanic mothers.^13,14^ Determinants of these disparities include differences in prenatal breastfeeding intention (with Hispanic mothers having higher intention than Black and White mothers),^13,15^ receipt of education, and structural barriers, including lack of or insufficient insurance coverage for high-quality breast pumps and paid parental leave.^13,16,17^ Furthermore, prolonged mother-infant separation and varied neonatal intensive care unit (NICU)–specific lactation support (eg, access to NICU-trained lactation support personnel), can specifically affect mother–preterm infant dyads.^18^ National trends of initiation and continuation of mother’s milk provision are not well described among preterm infants. Existing data, limited to state-level analyses or quality improvement databases focused on the very low birth weight population and typically within the inpatient setting, have identified racial and ethnic disparities in mother’s milk provision.^5,8,19,20,21^ However, the extent of these lactation disparities on a national level among all preterm infants is unclear, especially in continuation after hospital discharge. Furthermore, it is not known whether there have been changes in mother’s milk provision among the preterm population during a period that saw wide-reaching public health recommendations and quality improvement efforts. This study’s goal is to use a nationally representative population-based database to describe rates of mother’s milk initiation and continuation at 12 weeks after birth among preterm infants by maternal race and ethnicity.

Among mothers with preterm infants, we aimed to examine national prevalence and trends over time of mother’s milk initiation and continuation at 12 weeks after birth from 2009 to 2019 by maternal race and ethnicity (non-Hispanic Asian, non-Hispanic Black, Hispanic, and non-Hispanic White) and to examine prevalence and trends in mother’s milk outcomes according to maternal race and ethnicity by gestational age subgroups to understand mother’s milk provision among infants with varying levels of medical vulnerability. We also aimed to examine independent associations of maternal race and ethnicity and mother’s milk initiation and continuation.

## Methods

### Data Source

This cross-sectional study is a retrospective analysis of Pregnancy Risk Assessment Monitoring System (PRAMS) survey results. PRAMS is a multistate perinatal surveillance system with standardized data collection (mailed survey followed by telephone survey) established by the CDC and administered by state departments of health.^22^ We use the term *mother* throughout this article to remain consistent with PRAMS survey terminology. Mothers are surveyed 2 to 6 months post partum and report data on gestational age, birth weight, maternal and infant demographic factors, and breastfeeding behaviors. Surveys are subsequently linked with birth certificate data. Additional details about PRAMS and its methods have been described elsewhere.^22^ Stratified and weighted sampling of birth certificates by state ensure representation of a state’s full birthing population, including populations that may be otherwise less represented in national data. In this way, mothers of preterm infants and minoritized racial and ethnic groups have balanced representation.^22^ The Boston Children’s Hospital institutional review board determined that PRAMS data use was in the exempt category of research; thus, informed consent was not required, in accordance with 45 CFR §46. This study followed the Strengthening the Reporting of Observational Studies in Epidemiology (STROBE) reporting guidelines for cross-sectional studies.^23^

### Population

We included surveys completed from 2009 through 2019; we did not include available data in 2020 and 2021 owing to the outsized impact of the COVID-19 pandemic on breastfeeding behaviors.^24^ PRAMS responses represent approximately 83% of the population after weighting.^22^ Of 430 044 mothers with surveys completed in 2009 to 2019, we excluded dyads with full-term infants or infants with unknown gestational age; race and ethnicity that was missing, unknown, or other; and dyads where the infant died, was not living with the mother, or where this information was missing. In addition, we excluded dyads where the mother was surveyed less than 12 weeks post partum, had unknown breastfeeding behaviors, or unknown covariates of interest. This left 61 341 (unweighted) mothers for our sample (eFigure in Supplement 1).

### Measures

Mother’s milk initiation was defined as those who responded yes to the PRAMS question, “Did you ever breastfeed or pump breast milk to feed your new baby?” Mother’s milk continuation was identified as those who completed the survey 12 or more weeks post partum, and responded yes to the question, “Are you currently breastfeeding or feeding pumped breast milk to your new baby?” or reported breastfeeding for 12 or more weeks on the question, “How many weeks or months did you breastfeed or pump milk to feed your new baby?”

Self-report of maternal race and ethnicity was obtained from birth certificate data. We used these data to create race and ethnicity categories: non-Hispanic Asian (including other Asian, Chinese, Japanese, or Filipino; hereafter, Asian), non-Hispanic Black (hereafter, Black), Hispanic (any race), and non-Hispanic White (hereafter, White). We did not include other or multiracial because of small sample sizes (<3% of the sample when combined). Other variables of interest were obtained from birth certificate data, including infant gestational age, categorized by PRAMS as early preterm (≤27 weeks), moderate preterm (28-33 weeks), and late preterm (34-36 weeks), maternal age (≤19 years, 20-34 years, and ≥35 years), prepregnancy maternal insurance (non-Medicaid, Medicaid, or none), maternal education (0-8 years, 9-11 years, 12 years, ≥13 years), marital status (married vs other), cesarean delivery (yes or no), maternal diabetes during or before pregnancy (yes or no), parity (primiparous or multiparous), and infant plurality (singleton or multiple).

### Statistical Analysis

 Data were analyzed from February 2022 to June 2024. We compared dyad characteristics and mother’s milk outcomes (initiation and continuation at 12 weeks) by race and ethnicity, using χ^2^ analysis for categorical variables and *t* tests for continuous variables. Two-tailed *P* < .05 was considered significant. We calculated rates of mother’s milk initiation and continuation at 12 weeks by year within racial and ethnic groups and used logistic regression models with year of birth to test the significance of linear trends over time within groups. To assess change in disparities over time, we ran an interaction term with maternal racial and ethnic group and year. We subsequently examined associations of mother’s milk initiation and continuation at 12 weeks with racial and ethnic groups using a multivariable regression model adjusting for gestational age subgroup, year of birth, maternal age, insurance, education, marital status, mode of delivery, diabetes, parity, and plurality, because these factors are known to be associated with breastfeeding.^21,25,26,27^ We calculated crude and adjusted relative risks (RRs) using White dyads as the reference because they constituted the largest subgroup in our cohort. We used the NLMeans macro to generate RR from a ratio of event probabilities in our regression model.^28^ Finally, we calculated mother’s milk outcomes by racial and ethnic group among gestational age subgroups and performed logistic regression models with year of birth to assess linear trends over time. Analyses were conducted using SAS statistical software version 9.4 (SAS Institute), and accounted for the complex survey design using weights and stratification provided by PRAMS.

## Results

Among 1 523 131 weighted mother–preterm infant dyads, 5.1% of mothers (78 182 mothers) were Asian, 21.3% (323 956 mothers) were Black, 19.0% (289 770 mothers) were Hispanic, and 54.6% (831 222 mothers) were White. Additional population characteristics are described in Table 1. More Black dyads were represented in lower gestational age subgroups. Black and Hispanic mothers of preterm infants were less likely to be married, more likely to have nonprivate insurance, and more likely to be multiparous than Asian and White mothers.

**Table 1.** Characteristics of Mother-Preterm Infant Dyads by Self-Reported Maternal Race and Ethnicity From 2009 to 2019 US Pregnancy Risk Assessment Monitoring System Respondents

Characteristic	Mothers, No. (weighted %) (N = 1 523 131)			
	Asian (n = 78 182 [5.1%])	Black (n = 323 956 [21.3%])	Hispanic, any race (n = 289 770 [19.0%])	White (n = 831 222 [54.6%])
Gestational age category				
Extremely preterm (≤27 wk)	2984 (3.8)	23 715 (7.3)	12 928 (4.5)	26 014 (3.1)
Moderately preterm (28-33 wk)	15 883 (20.3)	81 002 (25.0)	63 272 (21.8)	165 945 (20.0)
Late preterm (34-36 wk)	59 315 (75.9)	219 329 (67.7)	213 571 (73.7)	639 263 (76.9)
Maternal age, y				
≤19	1542 (2.0)	27 575 (8.5)	29 630 (10.2)	41 863 (5.0)
20-34	53 400 (68.3)	242 875 (75.0)	200 655 (69.2)	636 142 (76.5)
≥35	23 241 (29.7)	53 506 (16.5)	59 485 (20.5)	153 216 (18.4)
Maternal education, y				
0-8	1657 (2.1)	3740 (1.2)	40 736 (14.1)	9210 (1.1)
9-11	4739 (6.1)	49 898 (15.4)	62 488 (21.6)	65 824 (7.9)
12	11 634 (14.9)	114 881 (35.5)	87 056 (30.0)	202 050 (24.3)
≥13	60 152 (76.9)	155 427 (48.0)	99 490 (34.3)	554 138 (66.7)
Married	67 342 (86.1)	83 606 (25.8)	132 783 (45.8)	564 325 (67.9)
Primiparous	35 733 (45.7)	115 641 (35.7)	96 360 (33.3)	356 526 (42.9)
Multiple birth	8653 (11.1)	34 217 (10.6)	24 758 (8.5)	121 719 (14.6)
Maternal insurance				
Medicaid	12 911 (16.5)	145 026 (44.8)	81 296 (28.1)	156 295 (18.8)
No insurance	7117 (9.1)	53 330 (16.5)	100 757 (34.8)	107 060 (12.9)
Cesarean delivery	36 123 (46.2)	159 085 (49.1)	132 396 (45.7)	402 450 (48.4)
Diabetes during or before pregnancy	17 869 (22.9)	37 220 (11.5)	50 292 (17.4)	93 137 (11.2)
Infant hospitalized at time of survey completion	1101 (1.4)	7311 (2.3)	4444 (1.5)	10 169 (1.2)
Infant sex				
Female	32 205 (41.2)	157 767 (48.7)	132 060 (45.6)	392 047 (47.2)
Male	45 977 (58.8)	166 189 (51.3)	157 711 (54.4)	439 174 (52.8)

**Figure 1.**
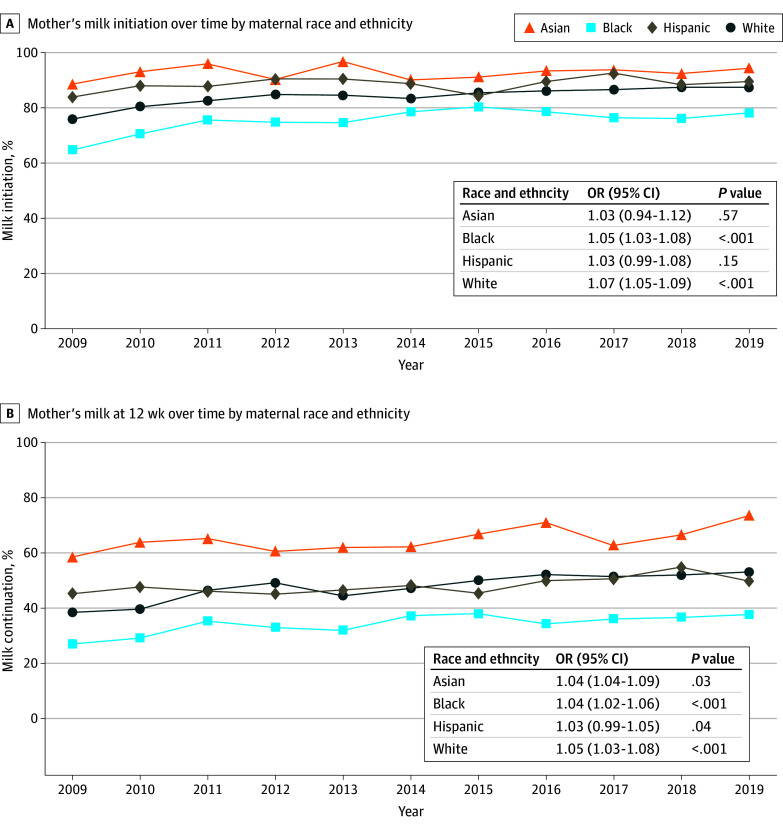
Rates of Mother’s Milk Initiation and Continuation at 12 Weeks Over Time by Maternal Race and Ethnicity Among Mothers With Preterm Infants (<37 Weeks’ Gestation) Odds of initiation increased significantly among Black and White mothers (*P* < .001), but not among Asian or Hispanic mothers (*P* > .10). Odds of mother’s milk continuation at 12 weeks increased significantly among all racial and ethnic groups (*P* < .05). OR indicates odds ratio.

From 2009 to 2019, mother’s milk initiation increased significantly for Black and White mothers but not for Asian or Hispanic mothers. Mother’s milk at 12 weeks increased significantly over time among all groups (Figure 1). We found significant differences in mother’s milk outcomes between racial and ethnic groups (Table 2). Overall, rate of mother’s milk initiation was highest for Asian mothers (92.8%; 95% CI, 91.1%-94.4%), followed by Hispanic (88.1%; 95% CI, 86.5%-89.8%), White (84.1%; 95% CI, 83.3%-84.9%), and Black (75.3%; 95% CI, 73.4%-76.6%) mothers. Mother’s milk at 12 weeks was highest for Asian mothers (65.4%; 95% CI, 62.7%-68.0%; a decrease of 27.4%), followed by Hispanic (48.2%; 95% CI, 46.1%-50.3%; a decrease of 39.9%), White (47.7%; 95% CI, 46.7%-48.7%; a decrease of 36.4%), and Black mothers (34.3%; 95% CI, 32.9%-35.7%; a decrease of 41.0%). On the basis of our interaction analysis, the disparity between Black and White mothers in mother’s milk provision did not significantly change over time. Black mothers had a significantly lower unadjusted risk of breastfeeding initiation (RR, 0.89; 95% CI, 0.88-0.91) and continuation (RR, 0.72; 95% CI, 0.67-0.75) compared with White mothers; however, after adjusting for covariates of interest, these differences attenuated to the null. Asian and Hispanic mothers had higher breastfeeding initiation and continuation compared with White mothers in unadjusted and adjusted models (Table 2). After adjusting for covariates of interest, when compared with White mothers, initiation remained higher for Asian (adjusted RR [aRR], 1.09; 95% CI, 1.06-1.12) and Hispanic (aRR, 1.10; 95% CI, 1.08-1.12) mothers, as did continuation (Asian mothers, aRR, 1.37; 95% CI, 1.24-1.47; Hispanic mothers, aRR, 1.33; 95% CI, 1.27-1.41).

**Table 2.** Overall US Prevalence and RR of Mother’s Milk Initiation and Continuation at 12 Weeks Among Mothers With Preterm Infants (<37 Weeks) According to Maternal Race and Ethnicity

Variable	Overall prevalence 2009-2019, % (95% CI)	RR (95% CI)	
		Unadjusted	Adjusted^a^
Mother’s milk initiation			
Asian	92.8 (91.1-94.4)	1.10 (1.08-1.13)^b^	1.09 (1.06-1.12)^b^
Black	75.3 (73.4-76.6)	0.89 (0.88-0.91)^b^	0.98 (0.96-1.00)
Hispanic, any race	88.1 (86.5-89.8)	1.05 (1.03-1.07)^b^	1.10 (1.08-1.12)^b^
White	84.1 (83.3-84.9)	1 [Reference]	1 [Reference]
Mother’s milk continuation at 12 wk			
Asian	65.4 (62.7-68.0)	1.37 (1.31-1.43)^b^	1.37 (1.24-1.47)^b^
Black	34.3 (32.9-35.7)	0.72 (0.67-0.75)^b^	0.98 (0.92-1.03)
Hispanic, any race	48.2 (46.1-50.3)	1.01 (0.96-1.06)	1.33 (1.27-1.41)^b^
White	47.7 (46.7-48.7)	1 [Reference]	1 [Reference]

Abbreviation: RR, relative risk.

^a^ Adjusted for gestational age category, year of birth, maternal prepregnancy Medicaid status, diabetes, multiparity, marital status, plurality, mode of delivery, maternal age, and maternal education.

^b^ *P* < .05.

**Figure 2.**
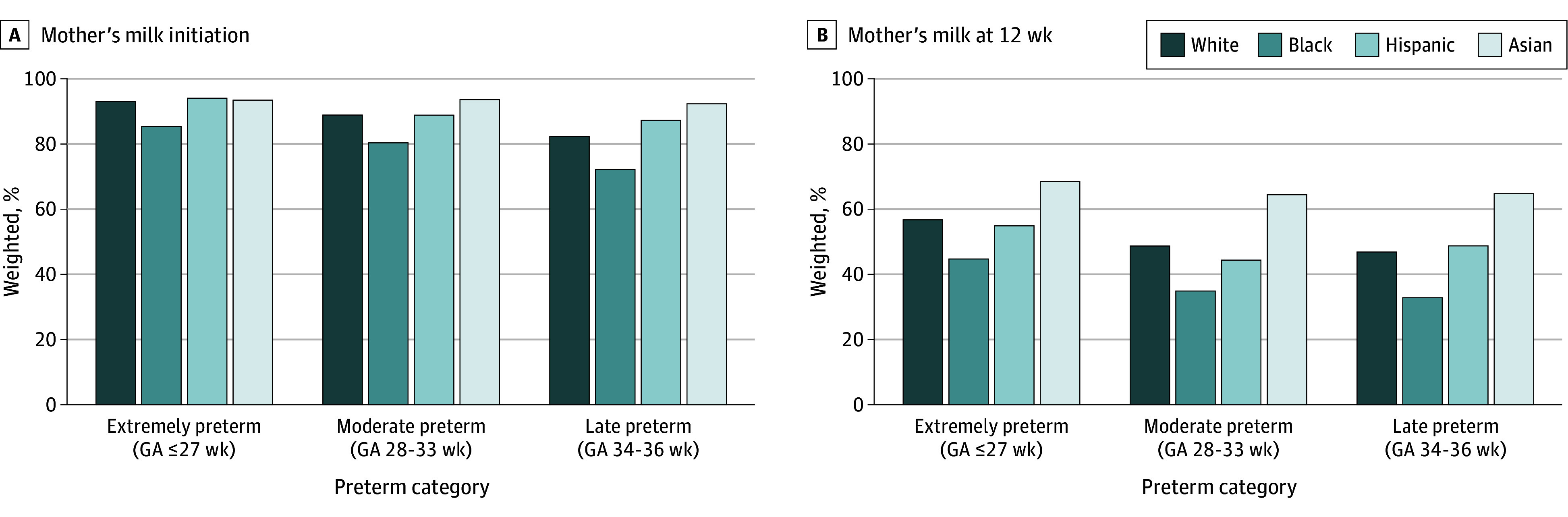
Period Prevalence of Mother’s Milk Initiation and Continuation at 12 Weeks by Maternal Race and Ethnicity, Shown by Gestational Age (GA) Subgroup

**Figure 3.**
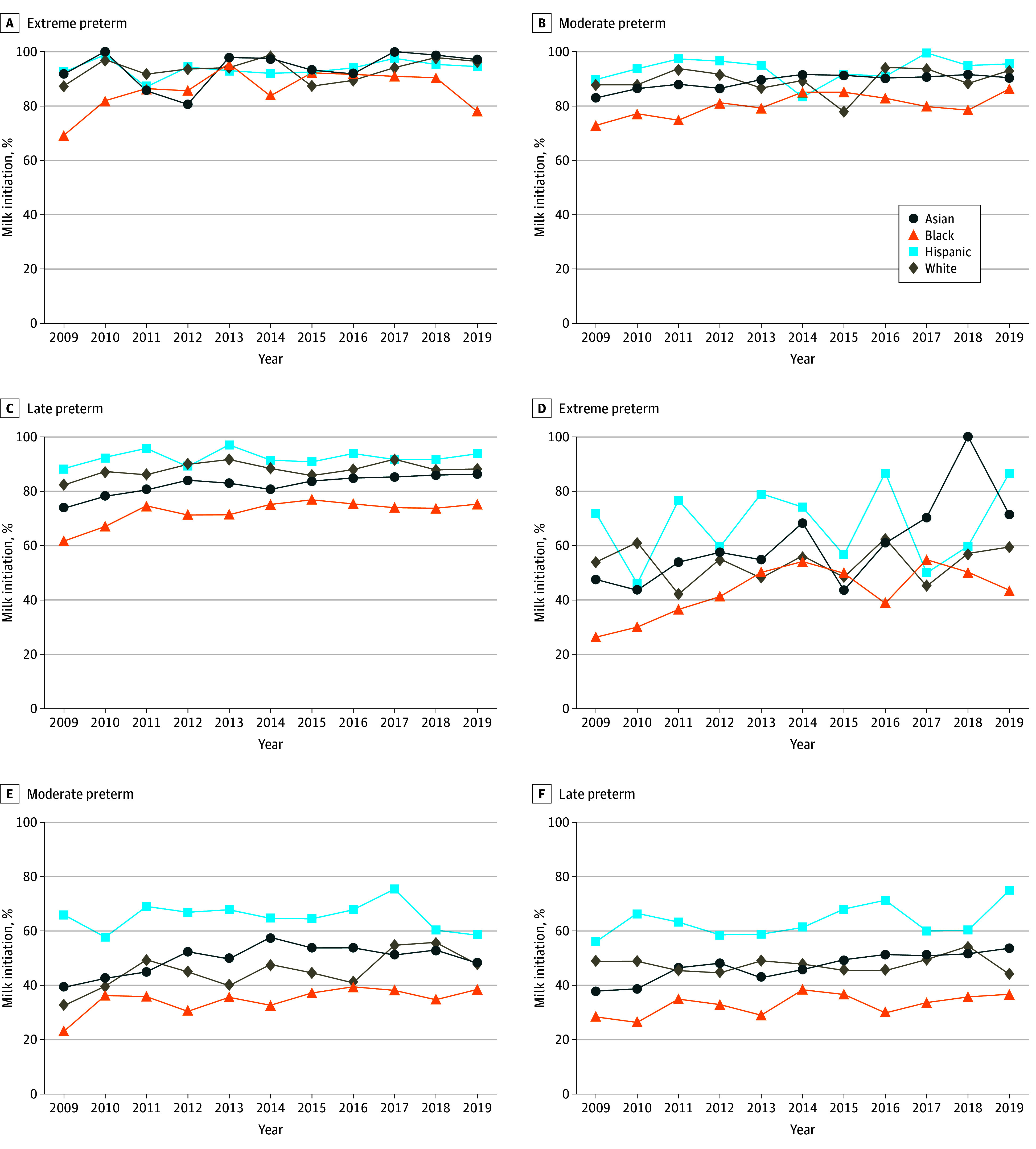
Rates of Mother’s Milk Initiation and Continuation at 12 Weeks Over Time by Maternal Race and Ethnicity in Gestational Age Subgroups Graphs show data for initiation (A-C) and continuation (D-F).

When examining racial and ethnic groups by gestational age subgroup, Black mothers had the lowest rate of mother’s milk initiation and continuation across all preterm gestational age subgroups (Figure 2). Among mothers of extremely preterm infants, Hispanic mothers had the highest rate of mother’s milk initiation (94.2%; 95% CI, 91.9%-96.6%) but a substantial decrease, with mother’s milk continuation of 55.0% (95% CI, 48.8%-61.3%) at 12 weeks. Asian mothers had the highest rate of mother’s milk initiation among moderate preterm and late preterm infants, and the highest rate of continuation in all gestational age subgroups. All racial and ethnic groups had highest rates of initiation and continuation among extremely preterm infants, followed by moderate preterm infants, with lowest rates in late preterm infants. Analysis of racial and ethnic groups by gestational age subgroup over time are displayed in Figure 3. All subgroups showed increased odds of mother’s milk provision from 2009 to 2019. In the early preterm population, Black (odds ratio [OR], 1.09; 95% CI, 1.03-1.15) and White (OR, 1.08; 95% CI, 1.02-1.14) mother-infant dyads had significant improvement in 12-week mother’s milk continuation. In the moderate preterm population, Black and White mother-infant dyads had significant improvement in both initiation (Black, OR, 1.06; 95% CI, 1.02-1.10; White, OR, 1.07; 95% CI, 1.03-1.11) and 12-week continuation (Black, OR, 1.04; 95% CI, 1.01-1.07; White, OR, 1.04; 95% CI, 1.02-1.07). In the late preterm population also, Black and White mother-infant dyads had significant improvement in initiation (Black, OR, 1.05; 95% CI, 1.02-1.08; White, OR, 1.07; 95% CI, 1.05-1.10) and 12-week continuation (Black, OR, 1.04; 95% CI, 1.01-1.06; White, OR, 1.06; 95% CI, 1.04-1.08). After adjustment, continuation was higher for Asian (aRR, 1.37; 95% CI, 1.24-1.47) and Hispanic (aRR, 1.33; 95% CI, 1.27-1.41) mothers compared with White mothers.

## Discussion

In this cross-sectional study, we used a nationally representative database to report trends in rates of mother’s milk initiation and continuation at 12 weeks by racial and ethnic group. From 2009 to 2019, mother’s milk outcomes increased over time in all groups; however, Black mothers had substantially lower rates of both mother’s milk initiation and continuation compared with other racial and ethnic groups, representing a persistent disparity in a modifiable factor known to offset substantial morbidities of prematurity and maternal health. This is especially notable because infants of Black mothers are disproportionately overrepresented in younger gestational age groups. Adjusting for sociodemographic and medical factors known to affect mother’s milk provision attenuated these findings to the null. This highlights the potential role of many modifiable contributors to this disparity. We also found that mother’s milk provision decreased between 27.4% and 41.0% from initiation until 12 weeks across racial and ethnic groups; however, this was least pronounced in extremely preterm dyads.

This study builds on previous local and multisite quality reports of racial and ethnic disparities in mother’s milk provision among the preterm population.^19,21,29,30^ Our findings that Black mothers have the lowest overall rates of mother’s milk provision compared with other racial and ethnic groups aligns with other studies. We were able to expand on these studies by examining mother’s milk initiation and continuation at 12 weeks, enabling a richer understanding of the decrease in mother’s milk provision during a period of medical vulnerability for preterm infants (birth hospitalization to home).^7^ Furthermore, the PRAMS database enabled a more complete understanding of national trends in racial and ethnic disparities across all preterm gestational age subgroups, which may inform targeted interventions.

Since 2009, there have been several public health efforts in support of breastfeeding among mothers of preterm infants.^8,31,32,33^ Quality efforts have focused on lactation promotion among the preterm population, but few have reported successful reduction or elimination in racial and ethnic disparities. Our results suggest no significant change in disparities over time among mother-preterm infant dyads. Reasons for this lack of reduction in disparities are multifactorial. Increasing evidence demonstrates racial and ethnic disparities in NICU care quality between and within hospitals.^20,29,34^ A recent study using the Vermont Oxford Network quality improvement database showed that Black infants are more likely to receive care at NICUs with lower composite quality measures compared with White infants.^35^ This suggests that lower quality of hospital-based lactation support could be a possible reason for lower overall rates of mother’s milk provision among Black mothers. Lactation support among mothers of preterm infants involves multiple, direct conversations with NICU staff. It is also possible that implicit or explicit bias,^36,37,38^ described among Black mothers in the NICU setting^39,40^ and when interacting with staff about breastfeeding,^41^ may influence provision of mother’s milk.

Other factors associated with decreased mother’s milk provision also occur more often among Black compared with White mothers, such as lower educational attainment, Medicaid insurance (a proxy for income), and adverse maternal medical factors (eg, diabetes and hypertension, which negatively impact lactation).^19,27^ Associations of maternal race and ethnicity and mother’s milk provision were attenuated to the null after adjusting for these factors, suggesting that these factors may explain, in part, the difference in mother’s milk among Black compared with White mothers. Higher maternal education enables more exposure to lactation education. Private health insurance and maternal education are associated with higher income, which improves access to jobs that support breastfeeding, through mechanisms including increased paid parental leave, dedicated time and space for pumping, access to high-quality breast pumps, and the ability to pay for child care.^16,17,18^

Mother’s milk provision not only has substantial benefits for the premature infant, but also for mothers, for whom longer lactation is associated with reduction in several postpartum complications (including metabolic disease and breast and ovarian cancer).^27^ Some studies also suggest that breastfeeding is associated with lower risk of postpartum depression.^42^ Rates of these complications are higher for Black mothers; therefore, the breastfeeding disparities demonstrated for Black mothers of preterm infants compounds to adversely affect lifelong health outcomes for the mother-infant dyad.^5,27,43,44^ Further efforts to address intersecting adverse social factors, particularly those mediated by systemic racism (eg, poverty and access to health care), breastfeeding education and support, and medical comorbidities, are needed to support Black mothers in lactation.^5,45,46^

Hispanic mothers of preterm infants had higher rates of mother’s milk provision than White mothers in this national study. Past population-level studies in very low birth weight infants have shown that in some areas of the US, provision of mother’s milk is higher among Hispanic mothers, whereas in other areas it is lower.^19,21,29,30^ Hispanic mothers in the US are a heterogeneous group, for whom nativity, cultural beliefs, and perceptions of formula are impactful in breastfeeding decision-making.^47^ Studies have also shown differences in NICU care quality among Hispanic and White mothers.^29,35^ NICU lactation support also may be different in these groups. For instance, language barriers in lactation support may impact lactation among Spanish-speaking Hispanic mothers.^48^ We also found higher rates of mother’s milk provision among Asian mothers, consistent with other literature.^5,7,21^ However, there is heterogeneity within Asian populations, and variation in breastfeeding among Asian mothers of full-term infants has been identified.^49^ We hypothesize that there may be similarly unappreciated disparities within Asian subpopulations. These findings emphasize the need for state and local analyses of specific barriers for mothers to initiate and continue mother’s milk for all preterm infants, as well as further stratification within racial and ethnic groups.^50,51,52^

A final important observation is that there remains a significant decline in rate of mother’s milk provision from birth to 12 weeks after birth among all racial and ethnic groups. This emphasizes the challenges in providing mother’s milk for preterm infants throughout the NICU hospitalization and potentially discharge home, a highly vulnerable period for continued mother’s milk provision, given competing priorities and systematic barriers for mothers to continue expressing milk or transition to direct breastfeeding, including return to work, presence of other children, and multiple medical appointments, among others.^53,54^

Strengths of this study include examination of mother’s milk provision among a national population-level sample of preterm infants with self-reported birth certificate race and ethnicity data, which are more accurate than hospital medical records. We also had a sufficient sample to examine gestational age subgroups over the course of 10 years.^55^

### Limitations

We acknowledge limitations of this work. First, although we examined 2 time points (initiation and continuation at 12 weeks), this study did not enable detailed longitudinal examination of mother’s milk provision over time or mother’s milk exclusivity. We did not have access to several factors that influence lactation, such as breastfeeding intent or self-efficacy, availability of paid parental leave, access to breast pumps, and family support for lactation. PRAMS also relies on mothers’ self-report of breastfeeding behaviors and duration, and recall bias may have occurred. However, accurate maternal recall of breastfeeding experiences has been demonstrated even remote from breastfeeding cessation.^56^ In addition, there is no specific distinction between donor breast milk (used in many NICUs for preterm infants) and mother’s own milk in the PRAMS survey, and the word *breastfeeding* may have been construed to include donor breast milk as well.

## Conclusions

Despite overall improvements in provision of mother’s milk initiation and continuation among preterm infants that mirror known increases among the healthy, full-term population, Black mothers have the lowest overall rates of mother’s milk provision compared with other racial and ethnic groups, but the disparity attenuates to the null after adjustment for several sociodemographic factors and medical comorbidities. Ongoing lactation support for Black mothers is needed, as mother’s milk provision is a modifiable factor to promote optimal health for preterm infants. Moreover, barriers to continued provision of mother’s milk for preterm infants should be identified and addressed.

## Notes

Supplement 1. **eFigure.** Exclusion flow diagram showing mother-infant dyads in study sample

Supplement 2.
Data Sharing Statement
