# Supplementary material for: Racial and Ethnic Disparities in Mother’s Milk Provision Among Mothers of Preterm Infants
Source: JAMA Netw Open. 2025 May 16;8(5):e2510781. doi: 10.1001/jamanetworkopen.2025.10781 (PMC12084848; doi:10.1001/jamanetworkopen.2025.10781)
Supplement: Supplement 1. — eFigure. Exclusion flow diagram showing mother-infant dyads in study sample [file jamanetwopen-e2510781-s001.pdf]

Supplemental Online Content

Kalluri NS, Cordova-Ramos EG, Hwang SS, Standish KR, Parker MG. Racial and ethnic disparities in mother's milk provision among mothers of preterm infants. *JAMA Netw Open*. 2025;8(5):e2510781. doi:10.1001/jamanetworkopen.2025.10781

eFigure. Exclusion flow diagram showing mother-infant dyads in study sample

This supplemental material has been provided by the authors to give readers additional information about their work.

© 2025 Kalluri NS et al. *JAMA Network Open*.

eFigure. Exclusion flow diagram showing mother-infant dyads in study sample.

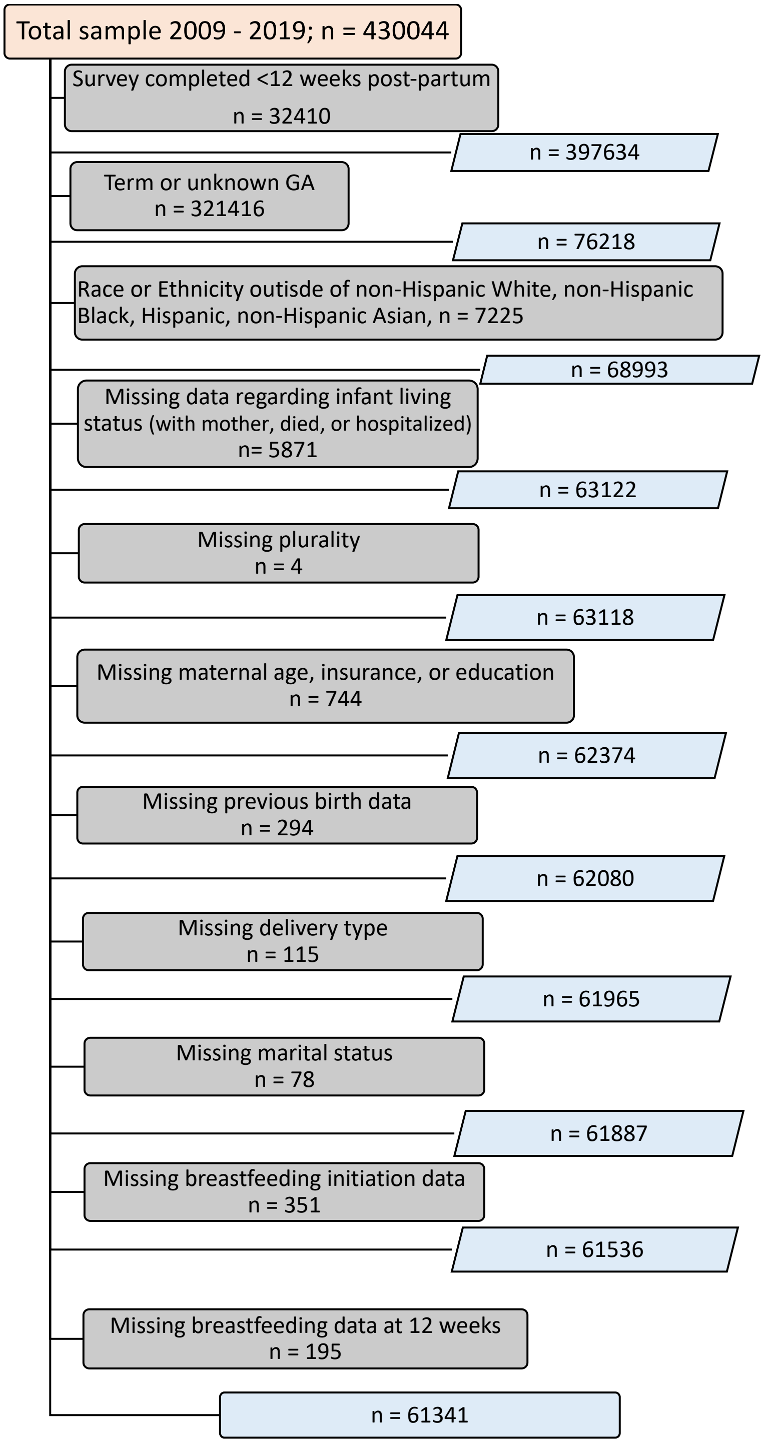

© 2025 Kalluri NS et al. *JAMA Network Open*.
