# Supplementary material for: Racial and Ethnic Disparities in Mother’s Milk Provision Among Mothers of Preterm Infants
Source: JAMA Netw Open. 2025 May 16;8(5):e2510781. doi: 10.1001/jamanetworkopen.2025.10781 (PMC12084848; doi:10.1001/jamanetworkopen.2025.10781)
Supplement: Supplement 2. — Data Sharing Statement [file jamanetwopen-e2510781-s002.pdf]

Data Sharing Statement

Kalluri. Racial and Ethnic Disparities in Mother's Milk Provision Among Mothers of Preterm Infants. *JAMA Netw Open*. Published May 16, 2025.
doi:10.1001/jamanetworkopen.2025.10781

Data
Data available: No

Additional Information
Explanation for why data not available: PRAMS data is available upon request to the CDC.
